# Complete genome sequence of a polyhydroxyalkaonate-accumulating bacterium, *Schlegelella aquatica* HS-12-14, isolated from a Korean hot spring

**DOI:** 10.1128/mra.01226-24

**Published:** 2025-01-27

**Authors:** Dariimaa Ganbat, Sondor Ganbat, Jae-Yoon Sung, Seong-Bo Kim, Dong-Woo Lee, Sang-Jae Lee

**Affiliations:** 1 Department of Bioscience and Research Center for Extremophiles and Marine Microbiology, Silla University 65486 , Busan, South Korea; 2 Department of Biotechnology, Yonsei University, Seoul, South Korea; 3 Bio-Living Engineering Major, Global Leaders College, Yonsei University, Seoul, South Korea; SUNY College of Environmental Science, Syracuse, New York, USA

**Keywords:** thermophiles, hot spring, polyhydroxyalkaonate, *Schlegelella aquatica*, genomes

## Abstract

We present the complete genome sequence of polyhydroxyalkaonate-accumulating moderately thermophilic *Schlegelella aquatica* HS-12-14 strain, isolated from a Korean hot spring. These findings contribute to valuable insights into the biosynthesis of polyhydroxyalkaonates in thermophiles and enhance understanding of *Schlegelella* strains.

## ANNOUNCEMENT

Polyhydroxyalkanoates (PHAs) offer a sustainable alternative to conventional polyesters, with the use of extremophiles for their production providing key advantages, particularly enhanced resistance to contamination (1–6). Among thermophiles, *Schlegelella*/*Caldimonas* spp. are especially promising (7, 8), and here we present the complete genome of *Schlegelella aquatica* HS-12-14, which will advance research into its PHA metabolism and biotechnological potential.

The strain HS-12-14 was isolated from Mageumsan hot spring (35°21′16.6″N 128°36′33.8″) water sample that was collected on 21 January 2022. An aliquot (1 mL) of the sample was mixed with 1 mL nutrient broth and incubated at 55℃ with shaking at 180 rpm for 24 h. Afterward, 500 µL of the culture was plated on nutrient agar and incubated overnight, and morphologically distinct colonies were selected and purified through sub-culturing. The strain was cryopreserved in a 5% dimethyl sulfoxide at −80℃. Taxonomic identification of the strain prior to genome sequencing was performed using 16S rRNA sequencing with universal primers 27F and 1492R. Sequence analysis using National Center for Biotechnology Information (NCBI) Basic Local Alignment Search Tool confirmed that strain HS-12-14 showed the highest 16S rRNA gene sequence identity (99.8%) to *Schlegelella aquatica* LMG 23380 (CP110257).

Genomic DNA was extracted using Wizard Genomic DNA Purification Kit (Promega) following the manufacturer’s protocols from 48 h culture incubated at 45℃ on nutrient agar plates. Genomic DNA was sequenced by PacBio Sequel II platform with Sequel Sequencing Kit v.3.0 following their standard workflow. The sequencing library was prepared using SMRTbell Express Template Prep Kit v.2.0 (Pacific Biosciences), and DNA was sheared using g-TUBE (Covaris) followed by size selection using AMPure PB beads (Beckman Coulter Inc.). The HiFi reads, generated through circular consensus sequencing analysis, were quality-filtered with a Q20 threshold, without adapter trimming process. This workflow generated 291,487 raw reads, totaling 3,492,200,401 bases (~1,018× coverage) with an average read length of 11,981 bp and an *N*_50_ length of 13,669 bp. Simultaneously, gDNA was sequenced on an Illumina NovaSeq 6000 instrument following their standard workflow for library preparation. This workflow uses Illumina TruSeq Nano DNA Sample Prep Kit for library preparation followed by adapter trimming by Trimmomatic v.0.38 (9). Quality control was performed using FastQC v.0.12.0 (10). The sequencing generated 150 bp paired-end reads, totaling 34,112,264 reads and 5,150,951,864 bp (~1.502× coverage).

Hybrid assembly of all read types was performed using Canu v.2.2 (11) and resulted a single consensus sequence of 3,429,320 bp with a 68.8% guanine and cytosine (GC) content. This chromosomal assembly was circularized using Circlator v.1.5.5 and rearranged to start at the *dnaA* gene as default (12). This sequence was annotated by the NCBI Prokaryotic Genome Annotation Pipeline v.6.7 (13) with default parameters, and it is predicted to contain 3,275 genes representing 3,138 protein-coding sequences, 2 complete sets of rRNA (5S, 16S, and 23S), 45 tRNAs, and 4 noncoding RNAs.

## Data Availability

This whole genome is indexed at GenBank under BioProject accession number PRJNA1175156. The complete genome sequence can be found under GenBank accession number CP171806. Raw sequencing data can be accessed via SRA numbers SRX26456404 and SRX26456405, and the 16S rRNA sequence is deposited under accession PQ556326.
